# Demethylase FTO inhibits the development of prostate cancer by upregulating EGR2 expression in an m6A manner

**DOI:** 10.55730/1300-0152.2629

**Published:** 2022-10-13

**Authors:** Zhenyu WANG, Huamin SUN, Hua ZHU, Donghua GU, Xinfeng CHEN, Yongsheng PAN, Bing ZHENG, Dongrong YANG

**Affiliations:** 1 Department of Urology, The Second Affiliated Hospital of Soochow University, Jiangsu, China; 2 Department of Urology, The Second Affiliated Hospital of Nantong University, Jiangsu, China; 3 Department of Food Safety and School Health, Nantong Center for Disease Control and Prevention, Jiangsu, China

**Keywords:** Prostate cancer, FTO, demethylase, EGR2

## Abstract

Fat mass and obesity-associated protein (FTO) is a demethylase and plays a vital role in various cancers. However, the regulation mechanism of FTO in prostate cancer (PCa) remains unclear. This study aimed to elucidate the mechanism of FTO in PCa. The function and mechanism of FTO-mediated in PCa were determined by gain-of-function assays and RNA-seq. We found that FTO expression in PCa tissues and two PCa cell lines were significantly lower than that in adjacent tissues and normal cell line. PCa cells after overexpression of FTO showed a significant lower in proliferation, migration, and invasion capabilities. RNA-seq displayed that FTO overexpression altered transcriptome landscape in Du145 and PC-3 cells, particularly upregulating EGR2 expression. FTO overexpression induced differential expression genes, including MYLK2, DNA2, CDK, and CDC (6, 7, 20, 25, and 45), which were mainly enriched in adjustment of cell cycle and growth pathways. Furthermore, FTO overexpression significantly reduced the EGR2 methylation level. Arresting the proliferation, migration, and invasion of Du145 cells induced by FTO overexpression was significantly rescued by EGR2 knockdown. FTO overexpression also significantly inhibited tumor growth and promoted EGR2 protein expression. Taken together, FTO suppresses PCa progression by regulating EGR2 methylation. We uncovered a novel regulatory mechanism of FTO in PCa and provide a new potential therapeutic target for PCa.

## 1. Introduction

Prostate cancer (PCa), a prevalent malignancy, presents high morbidity and mortality (Siegel et al., 2018). Early PCa can be completely cured, whereas the initial symptoms of PCa are not evident and developed rapidly. About 15% of patients that have just been diagnosed with PCa are found to have an advanced stage (Pronzato and Rondini 2005). Therefore, exploring the pathogenic mechanism of PCa and providing clinical treatment to lengthen the survival time of patients are necessary.

N6-methyladenosine (m^6^A) is a kind of dynamic posttranscriptional modification process on mRNA or noncoding RNA, which was coordinated by methyltransferase (termed as m6A writer), demethylase (termed as m6A eraser), and m6A-binding protein (termed as m6A reader). Previous studies have shown that m^6^A modifications regulate the splicing, translation, and stability of RNA, which are closely related to the progression of many diseases (Wang et al., 2018; Wu et al., 2018). For example, methyltransferase-like 3 can catalyze the occurrence of m^6^A modification to promote the development of lung cancer (Li et al., 2021), bladder cancer (Cheng et al., 2019), and colorectal cancer (Zheng et al., 2021). Therefore, analyzing the function and mechanism of m6A modification-related genes in PCa is of great significance to understand the occurrence and development of PCa.

Demethylase fat mass and obesity-associated protein (FTO) is an m6A eraser, which plays a vital role in the progression of various cancers (Chen and Du 2019). For instance, FTO demethylase facilitated active cell migration through m^6^A demethylation in lung adenocarcinoma (Ding et al., 2020). In bladder cancer, FTO promoted the proliferation of tumor cells in an m^6^A-dependent manner (Zhou et al., 2021). However, the regulation of FTO in the initiation and progression of PCa remains unknown.

In our study, we intended to explore the function and mechanism of FTO in PCa. We examined the expression of FTO between tumor tissues and normal tissues, elucidated the effect of FTO overexpression on PCa cells, screened out target genes of FTO, and demonstrated the relationship between them. This study might provide new insights into PCa tumorigenesis.

## 2. Materials and methods

### 2.1. Public database

The TNMplot database (https://www.tnmplot.com/) is a new setup of gene expression omnibus (GEO) (Bartha and Gyorffy 2021), which was used to analyze FTO expression in PCa samples in all available normal (n = 106) and tumor tissues (n = 283).

### 2.2. Clinical samples

A total of 24 pairs of PCa tumor tissues and adjacent noncancerous prostate tissues were obtained from 24 patients in our hospital. Freshly dissected tissues were fixed with 4% paraformaldehyde, embedded in paraffin, and then stored at room temperature. Our study was approved by the Ethics Committee of The Second Affiliated Hospital of Soochow University (S20210227-041). Informed consent was obtained from all patients prior to sample collection based on the guidelines of The Second Affiliated Hospital of Soochow University. All tissue samples were handled and made anonymous in accordance with the ethical and legal standards.

### 2.3. Immunohistochemical assays

All 24 pairs of PCa clinical samples collected were used for immunohistochemistry (IHC) and ROC analysis. Briefly, IHC was performed in 4-μm paraffin-embedded sections. These sections were deparaffinized in xylene and rehydrated in distilled water. Endogenous peroxidases were inactivated with 3% H_2_O_2_ for 10 min. Antigen retrieval was performed using citrate buffer. Sections were blocked with 5% BSA for 35 min and incubated with the primary antibody, FTO (proteintech# 27226-1-AP), at 4 °C overnight. Next, the sections were further incubated with secondary antibody for 35 min at room temperature. The target antigens were colored with diaminobenzidine (Maixin, Fuzhou, China), and the section was background counterstained using hematoxylin. After dehydration with ethanol and xylene, the slides were sealed and finally analyzed through light microscopy.

### 2.4. Cell culture and transfection

Human PCa cell lines, such as PC-3 (#CRL-1455™) and Du145 (#HTB-81), and normal human prostate epithelial cell, such as line RWPE-1 (#CRL-11609), were purchased from ATCC (Manassas, VA, USA). PC-3, Du145, and RWPE-1 cells were cultured in DMEM supplemented with 10% fetal bovine serum (FBS; #10099–141, Gibco) and 1% penicillin–streptomycin (#MA0110, Meilunbio) in a humidified atmosphere at 37 °C with 5% CO_2_.

For FTO overexpression, the full-length FTO sequence was reconstituted into the Plvx-EGFP-IRES-puro vector (Thermo Fisher Scientific, Inc.). The empty vector served as the control group. Next, the lentiviral vector of FTO and the empty vector were transfected into 293T cells for virus production using lipofectamine^®^ 2000 reagent (Invitrogen) according to the manufacturer’s instructions. Next, PC-3 and Du145 cells were infected with virus containing FTO vector for 48 h. Subsequently, stable cell clones of PC-3 and Du145 overexpressing FTO were screened by puromycin (#A1113802, Thermo Fisher) treatment.

For EGR2 silencing, three siRNAs (siRNA-220, siRNA-379, and siRNA-1318) and one siRNA-NC were designed against the EGR2 3′ UTR sequence and synthesized by GenePharm. Next, these siRNA sequences were transfected into Du145 cells using lipofectamine^®^ 2000 reagent (Invitrogen) according to the manufacturer’s instructions.

### 2.5. Real-time quantitative PCR (qRT-PCR) assay

In the qRT-PCR assay, each sample set had 3 repetitions. Total RNAs were extracted using a TRIzol reagent (Invitrogen, CA, USA). The concentration and purity of RNA samples were calculated using a Smart Specplus Spectrophotometer (BioRad). Next, cDNA was synthesized using the PrimeScript™ RT reagent kit (Takara). The qRT-PCR was performed using the SYBR^®^ Green qPCR Mix (Monad). The amplification procedure of qRT-PCR was 95 °C for 10 min following 40 cycles of 95 °C for 15 s and 60 °C for 60 s. The expression of each sample was normalized against that of GAPDH via the 2^−ΔΔCT^ method (Livak and Schmittgen 2001). The sequences of primers were validated from Primer-BLAST, which is shown in Table S1.

### 2.6. Western blot (WB)

Cells were lysed on ice using RIPA Lysis and Extraction Buffer (#89900, Thermo Fisher Scientific) for 15 min and centrifuged at 12000 rpm for 10 min at 4 °C. The supernatant was discarded, and protein concentration was determined by the BCA. Approximately 20 μg of protein was isolated by SDS-PAGE electrophoresis and transferred onto a PVDF membrane, and the transfer efficiency was checked with Ponceau red. The membrane was blocked at room temperature for 3 h with BST solution containing 5% skimmed milk. Next, membranes were incubated overnight with anti-FTO (Proteintech, 27226-I-AP, 1:1000) and anti GAPDH (Proteintech, 60004-1-Lg) at 4 °C and then with secondary antibodies, namely, Goat Anti-Rabbit IgG H&L(HRP) (Beyotime, A0208, 1:1000), Goat Anti-Mouse IgG H&L(HRP) (Beyotime, A0216, 1:1000) for 2 h. Afterward, the membranes were washed with TBST three times. Finally, bands were visualized using the ECL system.

### 2.7. CCK8 assay

Proliferation of PCa cells was detected using the Cell Counting Kit-8 (CCK8, Beyotime, China), following the manufacturer’s instructions. In brief, 100 μL of PC-3 and Du145 cells were seeded in 96-well plates at a density of 1 × 10^4^ cells/well and cultured at 37 °C with 5% CO_2_. After culturing for 0, 24, 48, and 72 h, 10 μL of CCK-8 solution was added into each well, and the cells were incubated for another 2 h. Finally, the absorbance of each well was measured at 450 nm.

### 2.8. Transwell assay

The Corning Transwell Kit (#3422, Corning, USA) was used for migration and invasion assessment. For Transwell migration, the assay was performed in a 0.8-μm 24-well plate chamber (#353097, FALCON), whereas the Transwell-invasion assay was performed in a 0.8-μm 24-well plate chamber with Matrigel (354480, BioCoat). Briefly, appropriately 500 μL of 5 × 10^4^ cell suspension was seeded in the upper chamber with 100 μL of serum-free medium, and 700 μL of 10% FBS was added into the lower chamber. After 48 h of incubation, cells were removed using cold PBS two times in the upper chamber and fixed with paraformaldehyde. Afterward, the chamber was stained with 0.2% crystal violet for 30 min at room temperature, sealed with neutral gum, and then imaged.

### 2.9. RNA-seq

Qualified RNA after removed rRNA extracted from PC-3 and Du145 cells was subjected to RNA-Seq. The cDNA libraries were constructed by using VAHTS Universal V6 RNA-seq Library Prep Kit for Illumina (NR604, Vazyme, China). Briefly, mRNA was fragmented and then reverse transcribed into first-strand cDNA followed by being synthesized into second-strand cDNA. Next, the purified double strand cDNA was end-repaired and dA-Tailing followed by adaptor ligation, and then a 150–200 bp insert fragment was obtained after two consecutive rounds of purification. Finally, the products were subjected to PCR amplification in enriched libraries. Qualified cDNA libraries were used for RNA sequencing performed on an Illumina Hiseq 2500 Genome Analyzer platform by Shanghai Yingbio Technology, Co., Ltd. Run parameters were set as pair-end with 150 bp. Subsequently, sequencing data was converted into raw data for quality control to remove low-quality data using Fast-QC (http://www.bioinformatics.babraham.ac.uk/projects/fastqc/). Afterward, clean data were de novo assembled to generate clean reads and then mapped into a reference genome (GRCh38) to obtain gene counts. Gene expression was normalized by fragments per kilobase million reads based on counts. Differentially expressed genes (DEGs) were evaluated using DESeq2 with a cutoff value of Log2FC > 1 or < −1 and FDR of < 0.05. DEG analysis of GO function and KEGG pathways was performed by KOBAS 2.0 server (Xie et al., 2011).

### 2.10. MeRIP-qPCR

MeRIP-qPCR is a method that measures methylation levels. To investigate the effect of FTO on the m6A modification level of EGR2 mRNA, the m6A modification of EGR2 mRNA was detected by MeRIP-qPCR after overexpression of FTO in PC-3 and Du145 cells. The RiboMeRIP™ m6A Transcriptome Profiling Kit (RIBBIO, China, C11051-1) was used to capture m6A-modified RNAs using the MeRIP method, according to the manufacturer’s procedure. Briefly, appropriately 18 μg of RNA samples was fragmented using a commercial kit (Invitrogen) and then incubated with anti-m^6^A antibody-bound magnetic beads (Millipore, USA). After washing the beads 3 times, the anti-m^6^A magnetic beads was mixed with MeRIP reaction liquid and incubated for 2 h at 4 °C. Next, the immunocomplex was eluted using the immunoprecipitation elution buffer and purified using the miRNeasy Mini Kit (QIAGEN). Finally, the eluted RNA was subjected to qRT-PCR detection.

### 2.11. Mice study

All mice experiments were carried out under the guidance of animal ethical management system. Male BALB/C nude mice (6-week-old, SPF grade) were provided by Shanghai Institute of Zoology, Chinese Academy of Sciences. Nude mice were randomly divided into two groups (with 5 mice each group): negative control group (NC group) and OE-FTO group. Suspensions of 2 × 10^7^ Du145 cells containing an FTO-overexpressed lentivirus vector or blank vector were subcutaneously injected into the left abdomen of nude mice. Tumor size was recorded every 3 days, and tumor volume was calculated as the formula: (length × width2)/2. Four weeks later, mice were euthanized, and tumors were resected and weighed.

### 2.12. HE staining

Fresh tumor tissue was fixed and embedded in paraffin and then cut into 5-μm sections. Sections were dewaxed, hydrated, routinely stained with hematoxylin solution for 5 min, and destained with 1% hydrochloric acid alcohol solution. Next, sections were stained with 0.5% eosin for 1 min. Afterward, the sections were dehydrated with ethanol, rinsed with xylene, mounted with neutral gum, and observed under an optical microscope (Olympus).

### 2.13. Statistical analysis

GraphPad Prism 9.0 was used for statistical analysis of data, and all data were expressed as mean ± standard deviation. Student’s t-test was used to compare the difference between two groups. One-way ANOVA following Tukey’s test was used to compare the difference among three or four groups. p < 0.05 indicated that the difference was statistically significant.

## 3. Results

### 3.1. FTO was downregulated in PCa tissue

**Figure 1:**
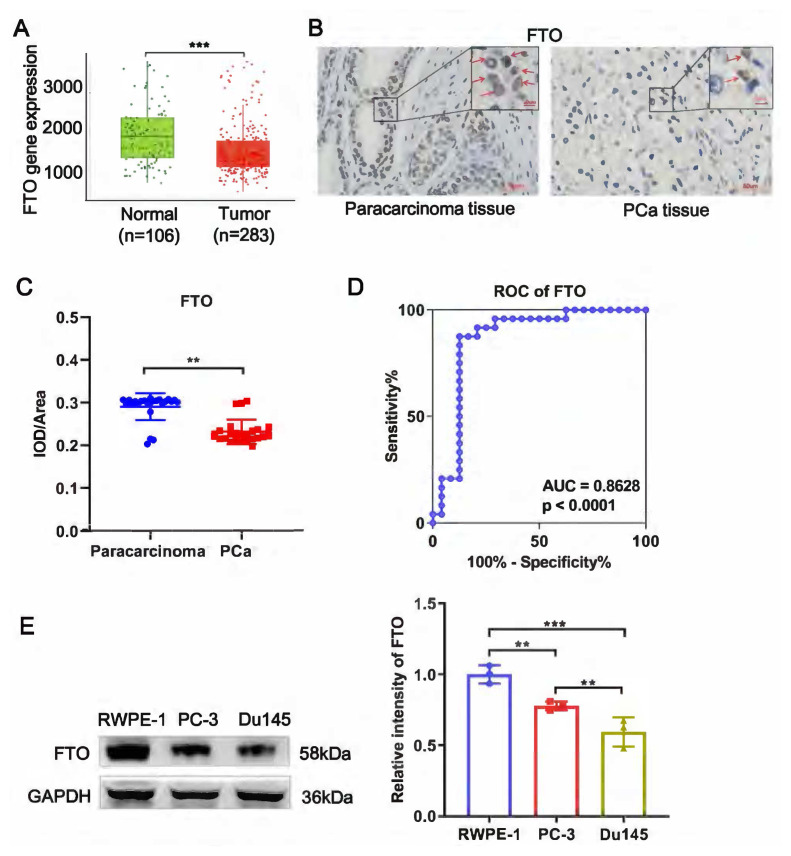
FTO was downregulated in PCa. (A) The expression of FTO in human PCa tissue was significantly lower than that in normal tissues based on the TNMplot database (p = 8.92e-08). (B) IHC staining (n = 24) of FTO in PCa tissues and their adjacent noncancerous tissues. (C) Average IOD/area of FTO in IHC staining. IOD/area = Integrated optic density/positive areas. (D) ROC curve for FTO. (E) FTO expression in PCa cell line (Du145 and PC-3) and normal cell of RWPE-1 detected by WB. ** p < 0.01, *** p < 0.0001.

To determine the expression pattern of FTO in PCa, we analyzed FTO expression in the TNM plot database with 329 PCa tumor samples and 106 normal samples. We found that FTO expression was reduced significantly in PCa tumor samples (Figure 1A). Next, we detected the expression of FTO in 24 pairs of clinical PCa and adjacent tissues. The IHC results showed that the positive areas for FTO staining were clearly observed in the paracancerous tissue, whereas a weaker expression was observed in PCa tissues (Figure 1B). IHC analysis was quantified using the integral optical density (IOD) method (Wang et al., 2003), and the result showed that FTO expression significantly decreased in PCa tissue (0.231) compared with paracancerous tissues (0.290, Figure 1C). The ROC curve based on FTO expression revealed that the area under curve was 0.86 (p < 0.0001) with 87.50% sensitivity and 83.33% specificity (Figure 1D), indicating that FTO may be a novel biomarker of PCa. These results concluded that FTO was lowly expressed in PCa, which may have a prognostic value. In addition, we determined the expression pattern of FTO in PCa cell lines (Du145, PC-3) and normal cell lines (RWPE-1). WB demonstrated that RWPE-1 has the highest FTO protein expression, followed by PC-3 cells, whereas Du145 cells exhibited the lowest FTO expression (Figure 1E). Therefore, the Du145 cell line was used for the exogenous manipulation of FTO overexpression.

### 3.2. FTO overexpression inhibited the proliferation, invasion, and migration of PCa cells

**Figure 2:**
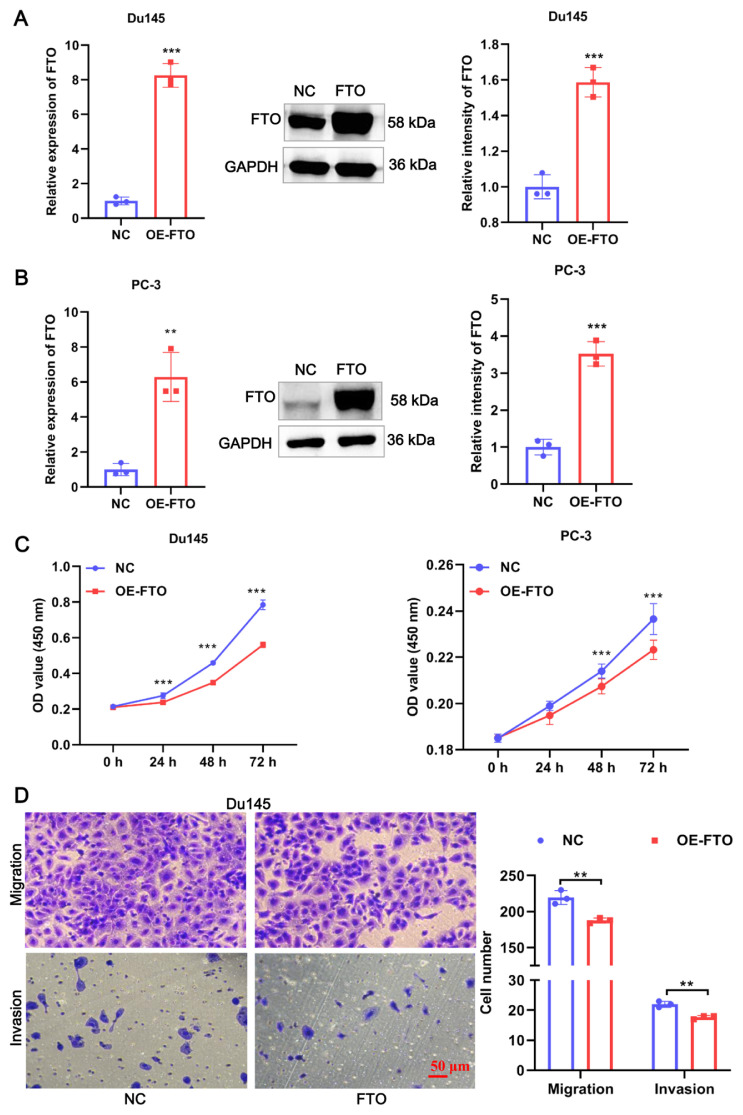
FTO inhibited the proliferation, invasion, and migration of PCa cells. (A) Transfection efficiency of FTO in Du145 cells was verified by qRT-PCR and WB. (B) Transfection efficiency of FTO in PC-3 cells was verified by qRT-PCR and WB. (C) Cell proliferation was measured by using CCK8 on Du145 cells and PC-3 cells. (D) Cell migration and invasion abilities were detected by Transwell on Du145 cells. ** p < 0.01, *** p < 0.0001.

To explore the role of FTO in PCa, we established the FTO overexpression of PCa cells and investigated the change in proliferation, migration, and invasion. The OE-FTO vector significantly enhanced FTO expression in PC-3 and Du145 cells both at mRNA and protein levels confirming the good overexpression efficiency (Figures 2A and 2B). Subsequently, we found that overexpression of FTO significantly suppressed the proliferation ability of both PC-3 and Du145 cells (Figure 2C). Similarly, the migration and invasion abilities of Du145 cells were significantly inhibited by FTO overexpression (Figure 2D). Curiously, the migration and invasion were barely detectable in PC-3 cells by Transwell assay. Regardless, these results suggested that FTO overexpression inhibited proliferation, migration, and invasion of PCa cells.

### 3.3. Analysis of DEGs regulated by FTO

**Figure 3:**
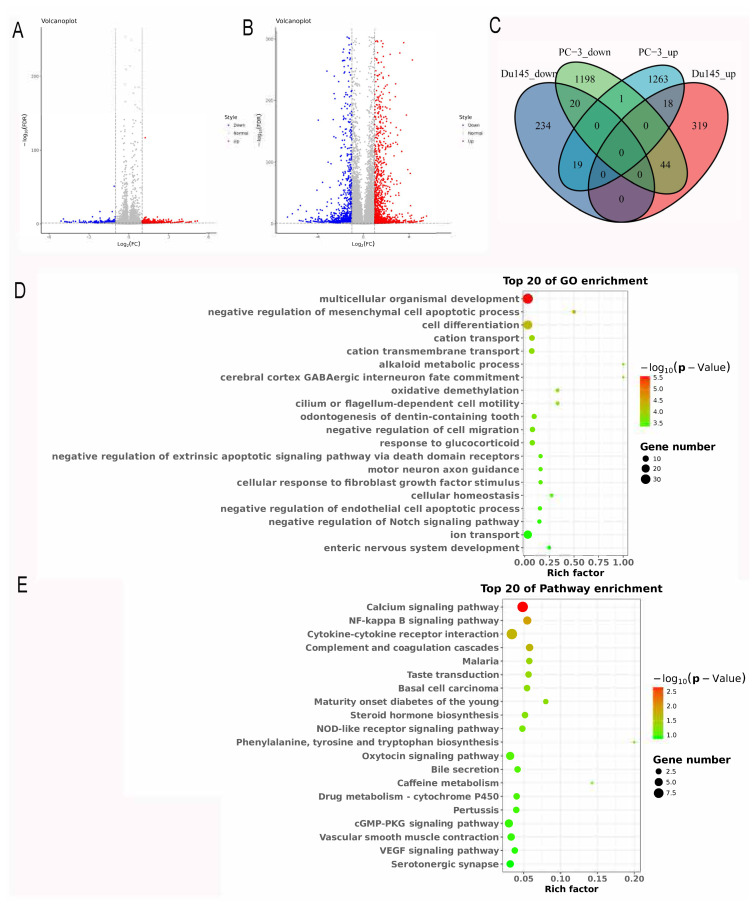
Analysis of the DEGs regulated by FTO. (A) DEGs between the NC group and OE-FTO in Du145 cells. (B) DEGs between NC and OE-FTO in PC-3 cells. DEGs screened using DESeq2. The upregulated DEGs with a fold change > 2 and FDR < 0.05 were marked in red. The downregulated DEGs with a fold change < 0.5 and FDR < 0.05 were marked in blue. (C) Venn diagram of DEGs in Du145 and PC-3 cells. (D) Top 20 enriched GO terms of FTO-associated DEGs in Du145 cells. (E) Top 20 enriched KEGG pathways of FTO-associated DEGs in Du145 cells.

To explore potential molecules in PCa cells that respond to FTO overexpression, we characterized the transcriptome landscape in PC-3 and Du145 cells after FTO overexpression. A total of 654 DEGs were identified in Du145 cells, including 381 upregulated DEGs and 273 downregulated DEGs in the OE-FTO group, compared with the NC group (Figure 3A). A total of 2564 DEGs were identified in PC-3 cells, including 1301 upregulated DEGs and 1263 downregulated DEGs in the OE-FTO group compared with the NC group (Figure 3B). In addition, 101 overlapping DEGs were shared between PC-3 and Du145 cells, of which 18 were upregulated DEGs and 20 were downregulated DEGs in both cell lines (Figure 3C).

**Figure 4:**
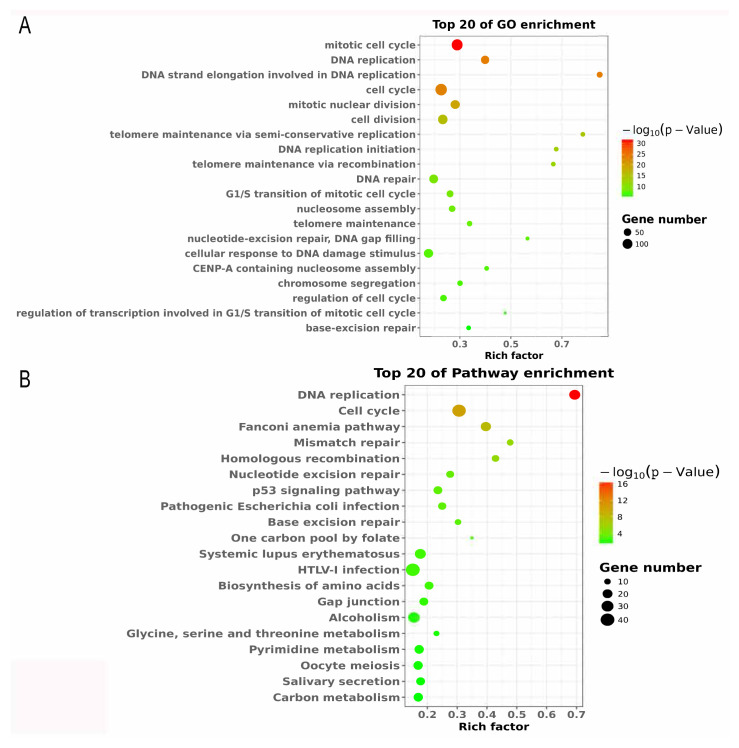
Analysis of DEGs in PC-3 cells regulated by FTO. (A) Top 20 enriched GO terms of FTO-associated DEGs in PC-3 cells. (B) Top 20 enriched KEGG pathways of FTO-associated DEGs in PC-3 cells.

Next, all DEGs were subjected to GO and KEGG pathway analysis using KOBAS 2.0 server. GO enrichment analysis showed that these DEGs in Du145 cells were mainly enriched in oxidative demethylation, negative regulation of cell migration, and negative regulation of mesenchymal cell apoptotic process, implicating that DEGs may be involved in molecular functions related to methylation and cellular growth (Figure 3D). Moreover, KEGG pathway analysis showed DEGs in Du145 cells, including calmodulin-like 6 (CALML6) and myosin light chain kinase 2 (MYLK2), which were mainly integrated into cellular growth-related pathway, such as calcium, NF-kappa B, NOD-like receptor, cGMP-PKG, and VEGF signaling pathway (Figure 3E). DEGs in PC-3 were mainly enriched cellular growth and cell cycle-related function of GO terms, such as mitotic cell cycle, DNA replication, cell cycle, etc. (Figure 4A). DEGs in PC-3 cells, including DNA replication helicase/nuclease 2 (DNA2), cyclin-dependent kinase family (1 and 2) and cell division cycle family (6, 7, 20, 25, and 45) were also involved in the cellular growth-related pathway, such as DNA replication, cell cycle, p53 signaling pathway, and mismatch repair pathway (Figure 4B). These results indicated that these DEGs participated mainly in adjustment of cell proliferation, cell cycle, and cell growth.

### 3.4. FTO reduced EGR2 methylation and promoted EGR2 expression

**Figure 5:**
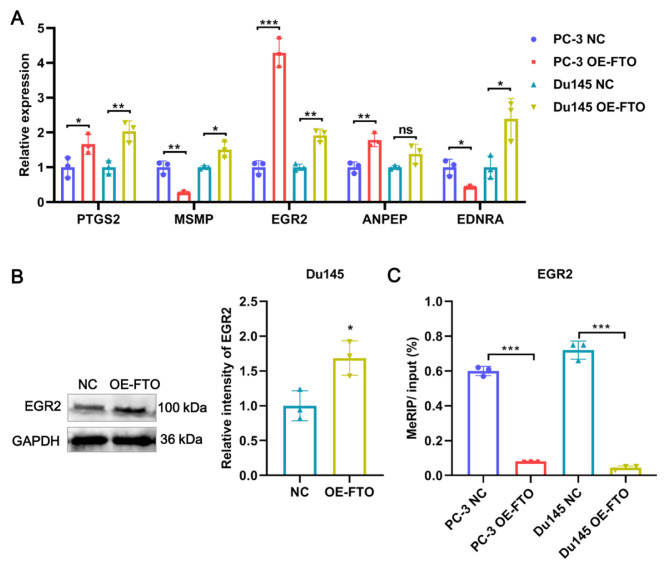
FTO reduced EGR2 methylation and promoted EGR2 expression. (A) qRT-PCR validation results for 5 candidate genes. (B) The relative expression of EGR2 was detected by WB in FTO-overexpressed Du145 and PC-3 cells. (C) Methylation levels of EGR2 were determined using MeRIP-PCR in Du145 cells after FTO overexpression. * p < 0.05, ** p < 0.01, *** p < 0.0001.

Subsequently, to verify the reliability of RNA sequencing results, 5 DEGs (ANPEP, EGR2, PTGS2, EDNRA, MSMP) with high expression abundance and m6A site were selected for qRT-PCR verification. The results showed that EGR2 and PTGS2 were significantly upregulated in the OE-FTO group in Du145 and PC-3 cells compared with the NC group, whereas the expression of the remaining three DEGs did not show a consistent trend in two PCa cell lines (Figure 5A). In addition, FTO overexpression had a more dramatic effect on EGR expression, particularly in PC-3 cells, causing a 4-fold upregulation, and 2-fold upregulation in Du145 cells (Figure 5A). Hence, we focused on EGR2 for subsequent experiments. The results of WB showed that FTO overexpression resulted in the upregulation of protein expression of EGR2 in Du145 cells (Figure 5B), which was consistent with qRT-PCR. Next, we measured the m^6^A modification level of EGR2 after FTO overexpression in Du145 cells. The MeRIP-qPCR results demonstrated that FTO overexpression significantly decreased the m6A enrichment on EGR2 compared with the NC group both in Du145 and PC-3 cells, suggesting that EGR2 methylation level was reduce after FTO overexpression (Figure 5C). Given that, methylation as an important posttranslational modification could affect gene expression (Liu et al., 2019). Therefore, we inferred that EGR2 is a demethylation target gene of FTO, and FTO promotes the expression of EGR2 by reducing the m^6^A level.

### 3.5. FTO suppresses PCa progression by regulating EGR2 in vitro

**Figure 6:**
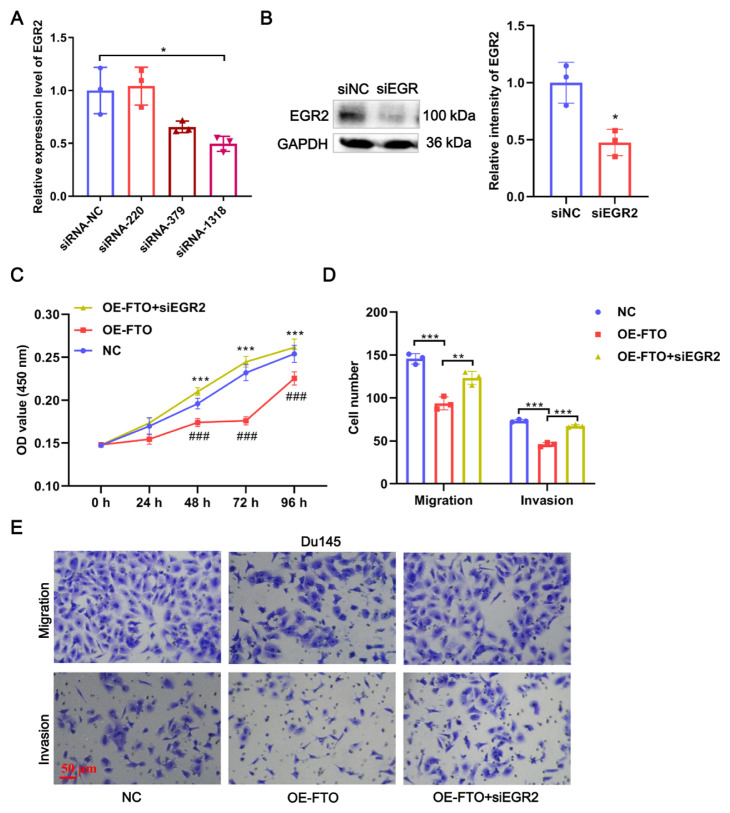
FTO suppresses PCa progression by regulating EGR2 in vitro. (A) Knockdown of EGR2 in Du145cells by 3 siRNA fragments (siRNA-220, siRNA-379, and siRNA-1318). The interference efficiency of siEGR2 was validated by qRT-PCR. (B) The interference efficiency of siEGR2 (siRNA-1318) was validated by WB. (C) EGR2 knockdown rescued the proliferation of Du145 cells suppressed by FTO overexpression, which was measured by CCK8. (D) EGR2 knockdown rescued the migration and invasion of Du145 cells suppressed by FTO overexpression, which was measured by Transwell. (E) Representative images of Du145 cells in Transwell. * p < 0.05, ** p < 0.01, *** p < 0.0001.

To clarify whether anti-PCa effect regulated by FTO is mediated through enhancing EGR2 expression, we conducted rescue assays. Three siRNAs (siRNA-220, siRNA-379, and siRNA-1318) against EGR2 were designed, of which siRNA-1318 displayed the maximal interference efficiency when compared with the control (Figure 6A). Meanwhile, the protein expression of EGR2 was also significantly inhibited by siRNA-1318 (Figure 6B). Thus, the siRNA-1318 fragment was used for subsequent experiments. Furthermore, CCK8 assays revealed that the overexpression of FTO significantly inhibited proliferation when compared with the NC group, whereas these suppression effects were rescued by simultaneous siRNA-1318 of EGR2 (Figure 6C). Similarly, the knockdown of EGR2 significantly attenuated the inhibitory effects of FTO overexpression on the migration and invasion of Du145 cells (Figures 6D and 6E). Collectively, FTO inhibits the PCa progression by promoting EGR2 expression through reducing m^6^A level.

### 3.6. FTO inhibited tumor growth of PCa through EGR2

**Figure 7:**
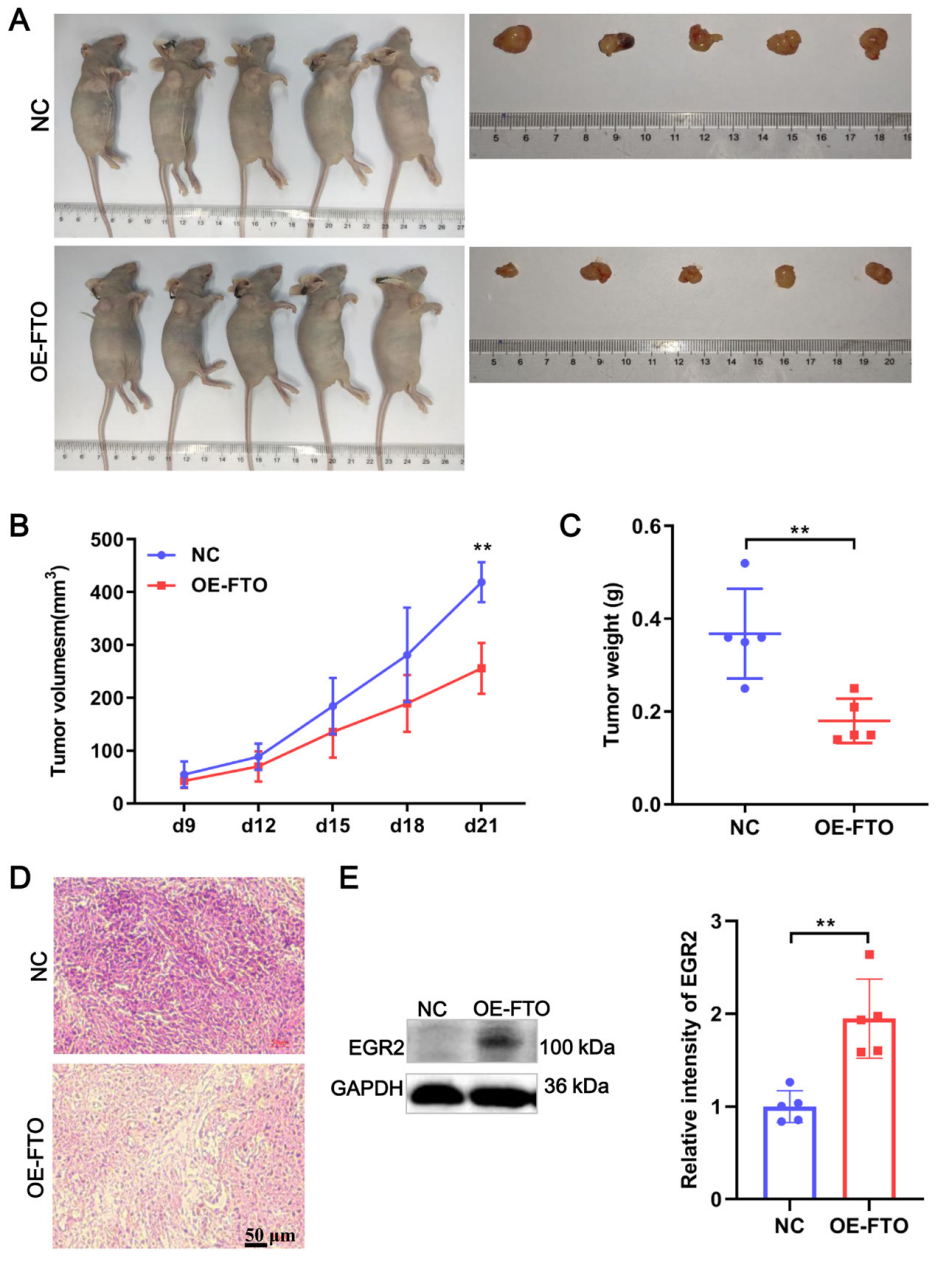
FTO-suppressed PCa tumor growth. (A) Images of tumor-bearing mice. Tumor growth for individual mouse in each group was presented. (B) Tumor volume of mice in NC and OE-FTO group. (C) Tumor weight of mice in NC and OE-FTO group. (D) Representative images of HE staining of tumor tissues. (E) EGR2 expression in tumor tissue was detected by WB. ** p < 0.01.

To further interrogate the role of FTO in vivo, we constructed a xenograft tumor mouse model through subcutaneously injecting FTO overexpression stable Du145 cells (Figure 7A). The results showed that FTO overexpression resulted in a decrease in tumor volume from day 0 to day 21 (Figure 7B) and significant weight loss (Figure 7C). The denser and more compact tumor cells in HE staining were observed in the NC group compared with the OE-FTO group (Figure 7D). To further clarify the regulation of FTO to EGR2 in vivo, we detected EGR2 expression in tumor tissue. Expectedly, EGR2 protein expression level was significantly increased after FTO overexpression in tumor-bearing mice (Figure 7E). These results demonstrate that FTO inhibited tumor growth of PCa through EGR2.

## 4. Discussion

**Figure 8:**
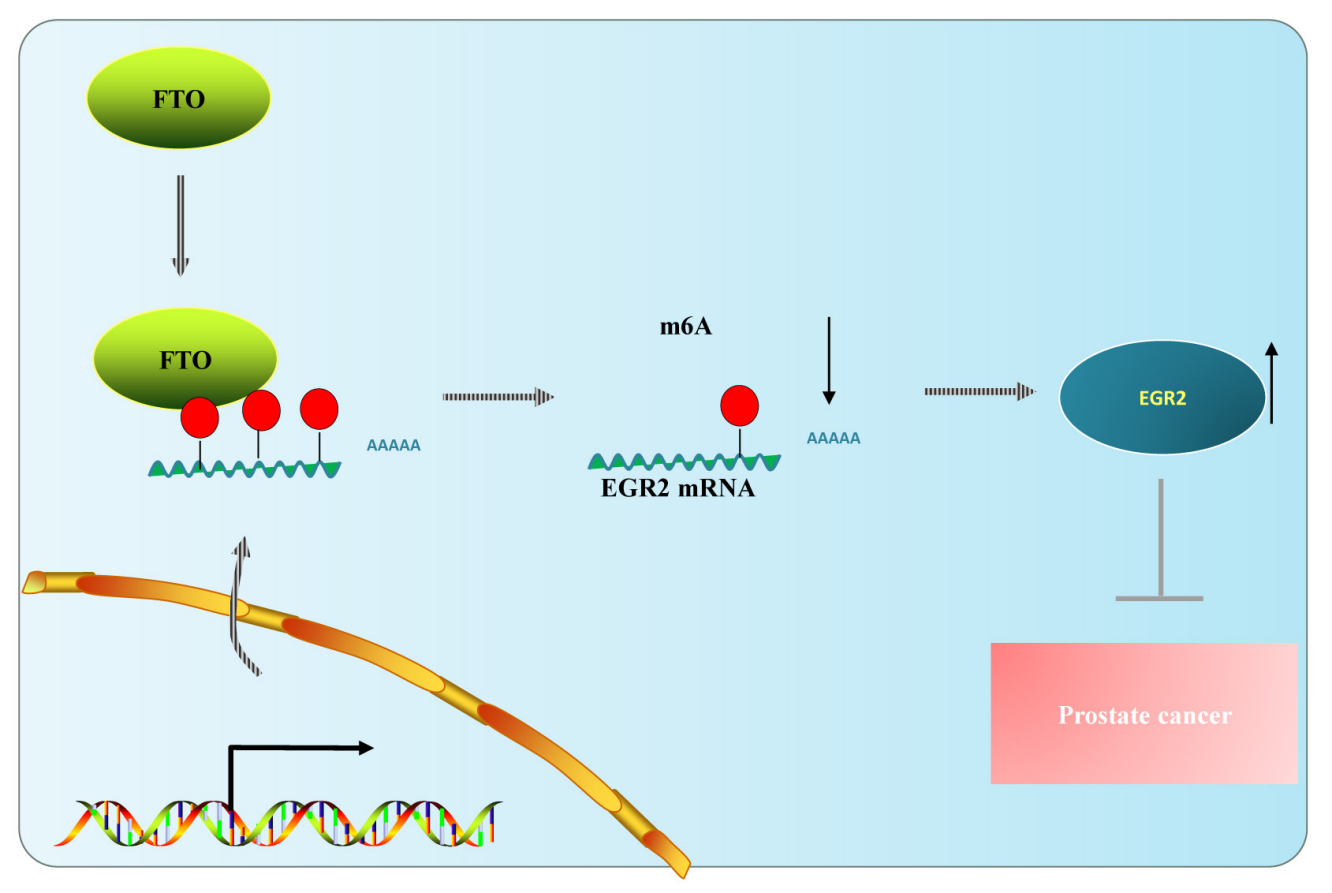
Schematic illustration of FTO-suppressed PCa progression. EGR2 is a demethylation target gene of FTO, and FTO promotes the expression of EGR2 by reducing the m6A level. FTO inhibited PCa development through EGR2 in vitro and in vivo.

Multiple studies have discovered that m^6^A modification controls cancer development by regulating the expression level of oncogenes and cancer suppressor genes. For example, the methyltransferase METTL-14 promotes the occurrence and development of leukemia via m^6^A RNA modification of MYB/myc (Weng et al., 2018). METTL3 represses the tumor suppressor SOCS2 expression in hepatocellular carcinoma through m^6^A regulatory mechanisms (Chen et al., 2018). In addition, few m^6^A modulators in PCa were examined in previous studies. In this study, we found that m^6^A demethylase FTO was downregulated in PCa in vitro and in vivo, and overexpression of FTO significantly inhibited the proliferation, migration, and invasion of PCa cells. RNA sequencing revealed that the overexpression of FTO led to a change in transcriptome landscape in PC-3 and Du145 cells. Mechanistically, FTO inhibits PCa development through EGR2, which is a demethylation target gene of FTO, and FTO promotes the expression of EGR2 by reducing the m6A level (Figure 8). Our study provides a mechanistical insight into the involvement of FTO-mediated EGR2 demethylation in the progression of PCa.

FTO, as the first identified m^6^A demethylase, becomes a research hotspot in cancer. FTO is well known to be involved in tumor progression by regulating m6A levels of target genes. For example, FTO restrains the proliferation, invasion, and metastasis of pancreatic cancer cells by reducing m^6^A levels of ring finger ubiquitin ligase 2 (Zeng et al., 2021). Moreover, FTO inhibits the tumor growth of clear cell renal cell carcinoma through decreasing the m^6^A level of PGC-1α mRNA (Zhuang et al., 2019). These studies suggest that FTO is an important factor in tumor progression. In addition, few reports on the regulation of m6A modification by FTO in PCa have been found. Zhu et al. proved that FTO suppresses the invasion and migration of PCa cells by regulating total m6A levels (Zhu et al., 2021) deeper demonstrated that FTO inhibits proliferation and metastasis of PCa through enhancing the stability of CLIC4 by reducing m6A level (Zou et al., 2022). Li et al. reported that FTO repression of PCa progression is dependent on MC4R m6A modification (Li and Cao 2022). Consistent with the abovementioned studies, we also proved that FTO overexpression significantly depressed the proliferation, migration, invasion, and tumor growth of PCa, but the difference is that demethylation target gene of FTO was EGR2. In short, FTO has the function of inhibiting PCa.

Here, we also found that EGR2 was involved in PCa progression. EGR2 is a member of a zinc finger transcription factor family, and it is widely involved in the occurrence and development of immune diseases (Morita et al., 2016; Taefehshokr et al., 2017) and tumors (Ahmed 2004). Previous reports have indicated that EGR2 could inhibit the metastasis of gastric cancer (Chen et al., 2019), regulate the growth and apoptosis of gastric cancer (Yang et al., 2021), and inhibit the tumor growth of thyroid carcinoma (Geng et al., 2021) and papillary thyroid carcinoma (Zang et al., 2020). These studies indicated that EGR2 plays a vital role in restraining tumor development. Interestingly, a study has also reported that EGR2 could increase IGF2BPs expression to enhance the m6A level of sphingosine-1-phosphate receptor 3 to accelerate tumorigenesis and metastasis of renal cell cancer (Ying et al., 2021). Moreover, the promoter transcriptional activity of EGR2 is silenced by epigenetic silencing complexes formed by miR-709 with H3K27me3 and Argonaute-1 (Adilakshmi et al., 2012). Long non-coding RNA EGR2-AS-RNA and EGR2 promoter antisense RNA could recruited H3K27me3, AGO1, AGO2, and EZH2 on the EGR2 promoter to suppressed EGR2 expression resulting in sciatic nerve injury (Martinez-Moreno et al., 2017; Moreno et al., 2022). Histone H2B monoubiquitination guided by EGR2 is required for peripheral nervous system myelination (Wüst et al.,2020). These results implicate the importance of EGR2 in epigenetics. However, as far as we know, there are few reports on the involvement of EGR2 in PCa, even less in an m6A-dependent manner. According to our study, upregulated FTO decreased EGR2 methylation level, which promoted the expression of EGR2 and inhibited tumor growth of PCa.

In addition, the novelty of this study deserves attention because we report for the first time that decreased EGR2 m^6^A level is responsible for FTO-suppressed PCa progression. However, this study also has several limitations. The first limitation is the effect of FTO on apoptosis, cycle, and other phenotypes of PCa cells, which has not been deeply explored. Another limitation is the molecular mechanism of the interaction between FTO and EGR2, which is not fully understood. Therefore, future studies will address these limitations.

## 5. Conclusion

We found that FTO was lowly expressed in PCa tissues and cell lines. FTO overexpression inhibited the proliferation, invasion, and metastasis, as well as altered the transcriptome landscape of PCa cells. As an m6A demethylase, FTO repressed the methylation level of EGR2 and promoted EGR2 expression. Moreover, disrupting the EGR2 expression partly rescued arresting of cell proliferation, migration, and invasion induced by FTO. Furthermore, FTO overexpression inhibited PCa tumor growth and EGR2 expression in vivo. Therefore, our findings provided novel insights into potential treatment methods of PCa.

## Supplementary

**Table S1:** Primer information used in this study.

Gene	Sequence (5′-3′)
GAPDH-F	ACAACTTTGGTATCGTGGAAGG
GAPDH-R	GCCATCACGCCACAGTTTC
FTO-F	AGTTATAGCTGTGAAGGCCCT
FTO-R	TGTCCCATGAGATCTTAAAACCA
ANPEP-F	TTCAACATCACGCTTATCCACC
ANPEP-R	AGTCGAACTCACTGACAATGAAG
EDNRA-F	TCGGGTTCTATTTCTGTATGCCC
EDNRA-R	TGTTTTTGCCACTTCTCGACG
EGR2-F	CCGGAGATGGCATGATCAAC
EGR2-R	GGTCAATGGAGAACTTGCCC
MSMP-F	CTGTGACACGTCCCAGCATC
MSMP-R	CCGAGGGTCAGATTTTTGCAC
PTGS2-F	ATGCTGACTATGGCTACAAAAGC
PTGS2-R	TCGGGCAATCATCAGGCAC
